# Influence of testing modality on bioefficacy for the evaluation of Interceptor^®^ G2 mosquito nets to combat malaria mosquitoes in Tanzania

**DOI:** 10.1186/s13071-022-05207-9

**Published:** 2022-04-11

**Authors:** Ummi Abdul Kibondo, Olukayode G. Odufuwa, Saphina H. Ngonyani, Ahmadi B. Mpelepele, Issaya Matanilla, Hassan Ngonyani, Noel O. Makungwa, Antony P. Mseka, Kyeba Swai, Watson Ntabaliba, Susanne Stutz, James W. Austin, Sarah Jane Moore

**Affiliations:** 1 Vector Control Product Testing Unit (VCPTU) Ifakara Health Institute, Environmental Health, and Ecological Sciences, P.O. Box 74, Bagamoyo, Tanzania; 2 Vector Biology Unit, Department of Epidemiology and Public Health, Swiss Tropical & Public Health Institute, Kreuzstrasse 2, 4123 Allschwil, Switzerland; 3 MRC International Statistics and Epidemiology Group, London School of Hygiene and Tropical Medicine, London, England; 4 Professional & Specialty Solutions, BASF SE, Public Health, 67117 Limburgerhof, Germany; 5 Professional & Specialty Solutions, BASF Corporation, Public Health Global Development, Research Triangle Park, NC 27709 USA; 6 University of Basel, Petersplatz 1, 4001 Basel, Switzerland; 7 Nelson Mandela African Institute of Science and Technology (NM-AIST), P.O. Box 447, Tengeru, Tanzania

**Keywords:** I-ACT, Ifakara ambient chamber test, ITNs, Chlorfenapyr, Bioefficacy, Bioassays

## Abstract

**Background:**

Insecticide-treated net (ITN) durability is evaluated using longitudinal bioefficacy and fabric integrity sampling post-distribution. Interceptor^®^ G2 was developed for resistance management and contains two adulticides: alpha-cypermethrin and chlorfenapyr; it is a pro-insecticide that is metabolized into its active form by mosquito-detoxifying enzymes and may be enhanced when the mosquito is physiologically active. To elucidate the impact of bioassay modality, mosquito exposures of the alphacypermethrin ITN Interceptor^®^ and dual adulticide Interceptor^®^ G2 were investigated.

**Methods:**

This study evaluated the performance of Interceptor^®^ G2 compared to Interceptor^®^ against local strains of mosquitoes in Tanzania. Unwashed and 20× times washed nets were tested. Efficacy of ITNs was measured by four bioassay types: (1) World Health Organisation (WHO) cone test (cone), (2) WHO tunnel test (tunnel), (3) Ifakara ambient chamber test (I-ACT) and (4) the WHO gold standard experimental hut test (hut). Hut tests were conducted against free-flying wild pyrethroid metabolically resistant *Anopheles arabiensis* and *Culex quinquefasciatus.* Cone, tunnel and I-ACT bioassays used laboratory-reared metabolically resistant *An. arabiensis* and *Cx. quinquefasciatus* and pyrethroid susceptible *Anopheles gambiae* sensu stricto and *Aedes aegypti*.

**Results:**

Against resistant strains, superiority of Interceptor^®^ G2 over Interceptor^®^ was observed in all “free-flying bioassays”. In cone tests (which restrict mosquito flight), superiority of Interceptor^®^ over Interceptor^®^ G2 was recorded. Mortality of unwashed Interceptor^®^ G2 among *An. arabiensis* was lowest in hut tests at 42.9% (95% CI: 37.3–48.5), although this increased to 66.7% (95% CI: 47.1–86.3) by blocking hut exit traps so mosquitoes presumably increased frequencies of contact with ITNs. Higher odds of mortality were consistently observed in Interceptor^®^ G2 compared to Interceptor^®^ in “free-flying” bioassays using *An. arabiensis*: tunnel (OR = 1.42 [95% CI:1.19–1.70], *p* < 0.001), I-ACT (OR = 1.61 [95% CI: 1.05–2.49], *p* = 0.031) and hut (OR = 2.53 [95% CI: 1.96–3.26], *p* < 0.001). Interceptor^®^ and Interceptor^®^ G2 showed high blood-feeding inhibition against all strains.

**Conclusion:**

Both free-flying laboratory bioassays (WHO Tunnel and I-ACT) consistently measured similarly, and both predicted the results of the experimental hut test. For bioefficacy monitoring and upstream product evaluation of ITNs in situ, the I-ACT may provide an alternative bioassay modality with improved statistical power. Interceptor G2^®^ outperformed Interceptor ^®^ against pyrethroid-resistant strains, demonstrating the usefulness of chlorfenapyr in mitigation of malaria.

**Graphical Abstract:**

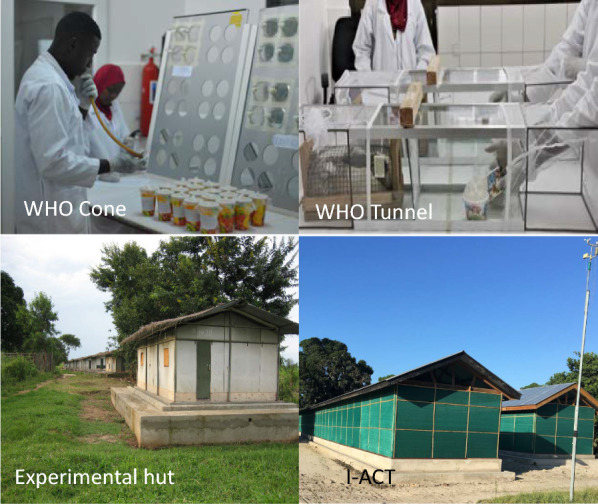

**Supplementary Information:**

The online version contains supplementary material available at 10.1186/s13071-022-05207-9.

## Background

As funding for malaria control falls and mosquito resistance to current public health insecticides increases, new and durable vector control tools that utilise new insecticide classes are needed [1]. For insecticide resistance management, a new Insecticide Treated Net (ITN) Interceptor^®^ G2 has been developed, coated with a mixture of the pyrethroid alpha-cypermethrin and the pro-insecticide chlorfenapyr [2]. Chlorfenapyr is an Insecticide Resistance Action Committee (IRAC) Group 13 insecticide, having a pyrrole chemistry that uncouples oxidative phosphorylation via disruption of the proton gradient (as a protonophore) to short circuit mitochondrial respiration through inner mitochondrial membranes of insect cells so that ATP cannot be synthesized, subsequently robbing insects of energy, resulting in death [3, 4]. Metabolic resistance is one of the main mechanisms of resistance observed in malaria vectors [5] where one or several detoxification gene families—cytochrome P450s (P450s), esterases and glutathione S-transferases (GSTs)—are overproduced to detoxify insecticides [6]. While this metabolism is a detoxification process, it can increase the potency of a pro-insecticide and may therefore be exploited as a means to control metabolically resistant insect populations [3]. The unique mode of action of chlorfenapyr on an insect’s metabolism is particularly relevant for the control of vectors harboring insecticide resistance mechanisms, as increased metabolic activity increases the conversion of the pro-insecticide into its potent n-dealkylated form and will consequently increase mosquito mortality [7].

For any new ITN to be used in public health, a thorough evaluation of its safety, efficacy and effectiveness is conducted based on World Health Organisation (WHO) standards and criteria [8]. The methods and scope of the laboratory tests and field trials required to attain WHO Prequalification (PQ) listing for ITNs are outlined in a set of WHO guidelines, last updated in 2013 [9]. As part of these guidelines, WHO recommends a set of standardised laboratory tests to ascertain the bioefficacy of pyrethroid ITNs, i.e., the ability of ITN products to kill, incapacitate (knock down) and prevent mosquitoes from blood-feeding. Laboratory bioefficacy tests are also a critical component of ITN durability evaluation used to confirm continued ITN bioefficacy after long-term use in the community [10]. The simplest and most commonly applied ITN bioefficacy test is the WHO cone bioassay where mosquitoes are held close to ITN in plastic cones and the number of mosquitoes knocked down (incapacitated) or killed is counted [9]. For ITNs with feeding inhibition mode of action, the WHO tunnel test is used, where a swatch of ITN with small holes (9 × 1-cm diameter) is made and is placed between mosquitoes and small animal bait overnight [11]. The WHO tunnel test has been shown to agree with experimental hut data, using laboratory-reared mosquitoes released into the huts [12]. Thresholds for the mosquito knockdown rate (95%) and mosquito mortality rate (80%) and blood-feeding inhibition (90%) using pyrethroid-susceptible *Anopheles* mosquitoes serve as performance benchmarks; candidate products are required to fulfill minimum performance standards, which have been established by WHO for qualification to be listed/recommended for their public health values to sustain and protect users from disease transmissions. This includes their physical durability and chemical contents recovered from nets replaced in the field over 3 years or as predicted from wash-resistance testing. For a net to be classified as a long-lasting ITN, i.e., LLIN, > 80% of nets tested should pass WHO cone/tunnel performance benchmarks after 3 years of use [13].

Cone tests are relatively easy to perform, high throughput and sensitive to detecting changes in bioavailable pyrethroids that act through rapid contact neurotoxicity. However, chlorfenapyr requires the mosquito to be metabolically and/or physiologically active (as it would be when encountering the ITN under user conditions) to bioactivate into the potent n-dealkylated form which elicits increased mosquito mortality. As mosquitoes are more metabolically active at night when flying and host-seeking during their typical circadian rhythms, the tunnel test may be more appropriate [14].

The President’s Malaria Initiative (PMI) currently uses WHO Tunnel tests for the durability monitoring of chlorfenapyr nets with 4 samples per net evaluated and 48 nets per sampling point [15]. This requires large numbers of mosquitoes and access to small animals for testing, so it cannot be done at all facilities in malaria-endemic areas. To accommodate high-throughput evaluation of whole ITNs for durability evaluation, the Ifakara ambient chamber test (I–ACT) was developed [16]. The Ifakara ambient chamber test (I-ACT) is not currently a recognized method approved by the WHO. However, it is currently seeking confirmation by direct comparison to approved methods. This is of particular importance where novel slow-acting chemistries cannot achieve the benchmark standards established for conventional pyrethroid ITN exposures yet may prove to be highly efficacious when tested according to their discrete modes of action. Like the experimental hut, I–ACT makes use of whole nets and human hosts to evaluate bioefficacy of field-used ITNs, but the assay is done under controlled conditions with laboratory-reared mosquitoes. Mosquitoes are released into net chambers within which the test net is hung with a volunteer sleeping beneath, and all mosquitoes are recaptured in the morning. The use of laboratory mosquitoes (rather than conducting experimental hut trials with wild mosquitoes) is done to improve the precision of estimates by releasing mosquito cohorts of a defined number of mosquitoes with high recapture rate (99%) at the conclusion of exposure intervals.

I-ACT bioassay has been used for evaluation of pyrethroid ITNs, was able to discriminate between products [17] and agreed with results of combined WHO cone and tunnel tests [16]. The I-ACT may show suitability for use in evaluation of pro-insecticides because: (i) the assay is run overnight, favouring malarial mosquitoes' circadian rhythms, (ii) the mosquitoes have a large arena to fly in, allowing them to be metabolically active, (iii) the mosquitoes have the opportunity to feed ad libitum and (iv) the I-ACT test eliminates infected mosquitoes that may represent a malaria transmission potential that cannot be completely excluded in WHO experimental huts—a significant safety benefit to volunteers. Therefore, the I-ACT method was evaluated alongside standard methods (i.e., experimental huts, WHO cone and tunnel tests) to provide direct evidence of its comparability. Interceptor^®^ (alpha-cypermethrin only) and Interceptor^®^ G2 (alpha-cypermethrin and chlorfenapyr) were evaluated to compare the performance of each assay for durability monitoring of pro-insecticidal ITNs.

## Methods

### Study area

The laboratory bioassays (I-ACT, WHO cone and WHO tunnel tests) were performed at the Vector Control Product Testing Unit (VCPTU) testing facility located at the Bagamoyo branch of Ifakara Health Institute (IHI), Tanzania (6.446º S and 38.901º E). The district experiences average annual rainfall of 800 mm–1000 mm, average temperatures between 24 ºC and 29 ºC and average annual humidity of 73%. The experimental hut study was conducted in Lupiro village (8.385° S and 36.673° E) in Ulanga District, southeastern Tanzania. The village is bordered by irrigated rice fields with average annual rainfall of 1200–1800 mm, average temperatures between 20 and 34 ºC and average annual humidity of 69%. The main malaria vector is *Anopheles arabiensis*, constituting > 99.9% of the *An. gambiae* complex species in the last test conducted in November 2020, and resistance to alphacypermethrin was recorded (57% mortality at 1 × WHO discriminating concentration) at the time of testing.

### Study design

The study was a five-arm comparative efficacy study to determine the performances of Interceptor^®^ G2 ITNs and Interceptor^®^ against pyrethroid-susceptible and -resistant mosquitoes measured by WHO cone bioassay, WHO tunnel test, Ifakara ambient chamber test (I-ACT) and experimental huts. Study arms were: (i) Interceptor^®^ G2, unwashed; (ii) Interceptor^®^ G2, washed 20 times; (iii) Interceptor^®^, unwashed; (iv) Interceptor^®^, washed 20 times; (v) SafiNet^®^ (negative control). The primary performance metric upon which the study was powered is 72-h mortality (M72), which is measured in all four bioassays. A secondary outcome was blood-feeding, which was measured in the three free-flying bioassays. Additionally, knockdown at 60 min (KD60) was measured in cone tests (Table 1).

**Table 1:** Outcomes measured in WHO cone bioassays, tunnel, I-ACT and experimental hut tests

	Cone bioassay	WHO tunnel test	I-ACT	Experimental hut
Primary outcome	72-h mortality	72-h mortality	72-h mortality	72-h mortality
Secondary outcome		Blood-feeding	Blood-feeding	Blood-feeding
Other outcomes	Knockdown at 60 min (KD60)			Deterrence^a^ and induced exophily^a^

^a^Not reported

### Mosquito test systems

IHI laboratory maintains local mosquito strains with resistant mechanisms present in the local population to avoid accidental release of new resistance alleles into the wild population. Four types of laboratory-reared mosquitoes with different resistance levels (confirmed at the time of testing) were used in the WHO cone, WHO tunnel and I-ACT tests: *Anopheles arabiensis* (Kingani strain, upregulation of cytochrome p450s, 14% mortality upon exposure to WHO discriminating doses of alpha-cypermethrin, which is reversed by piperonly butoxide (PBO) pre-exposure, which acts to block enzymatic detoxification mechanisms by inhibiting their metabolism), *Anopheles gambiae* s.s. (Kisumu strain, fully susceptible to all insecticide classes at WHO discriminating doses), *Aedes aegypti* (Bagamoyo strain, fully susceptible to all insecticide classes at WHO discriminating doses) and *Culex quinquefasciatus* (Bagamoyo strain, 6% mortality upon exposure to WHO discriminating doses of alpha-cypermethrin, which is partially reversed (only moderate susceptibility is restored from inhibition of detoxifying mechanism) by PBO pre-exposure). In I-ACT and tunnel tests, sugar-starved (8 to 9 h) nulliparous female mosquitoes, 5–8 days old, were used. For cone bioassay, 2–5-day-old nulliparous sugar-fed female mosquitoes were challenged. Laboratory colonies were maintained by feeding larvae Tetramin^®^ tropical fish food and adults on blood between 3 and 6 days after emergence and 10% sugar solution ad libitum. Temperature and humidity within the insectary are maintained between 27 ºC ± 5 ºC and 40%–100% RH, relatively following MR4 guidelines [18]. For the experimental hut assays, only wild populations of *An. arabiensis* and *Culex quinquefasciatus* were collected in sufficient numbers for evaluation.

### Test nets

ITNs were supplied by BASF in November 2019 and stored at optimal conditions (25 to 32 ºC) before testing and during the experimental phase. Interceptor^®^ G2 nets were from two different production batches (two batches were used for experimental hut only and one batch for other biossays) and Interceptor^®^ from one production batch. Interceptor^®^ G2 is made from 100-denier polyester coated with a mixture of wash-resistant formulation containing 200 mg/m^2^ chlorfenapyr and 100 mg/m^2^alpha-cypermethrin. Interceptor^®^ LN is made from 100-denier polyester coated with 200 mg/m^2^ alpha-cypermethrin. SafiNet is an untreated polyester net manufactured by A to Z Textiles Mills, Ltd., Tanzania, and was used as a control. The nets were washed 20 times according to a protocol adapted from the standard WHO washing procedure [9] using 20 g/l palm soap (Jamaa) and dried flat in a shaded area. The interval of time used between two washes (i.e. regeneration time) was 1 day for both Interceptor^®^ G2 and Interceptor^®^ ITNs.

### WHO cone bioassays

**Fig. 1:**
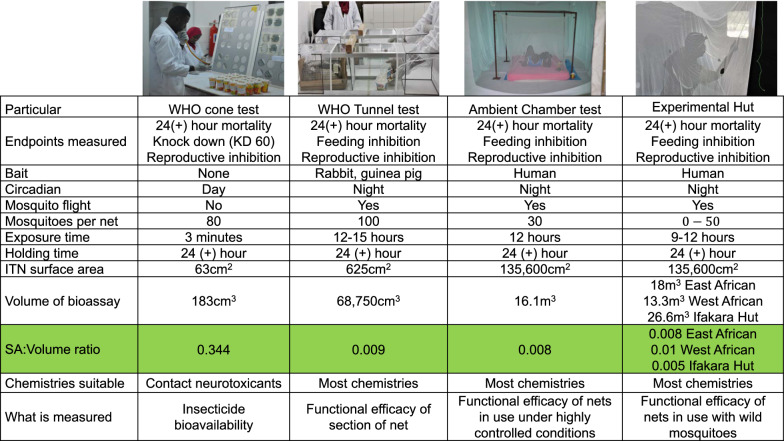
Critical factors for consideration for ITNs bioassays

WHO cone tests were performed between October 2020 and February 2021 according to standard WHO procedures [9], with two modifications: the test board was set at 60º tilt [19] and holes were cut in the boards (Fig. 1) to maximise mosquito contact with the test nets during exposures. From each treatment arm, three nets were randomly sampled, and five net swatches of 25 cm × 25 cm size were cut from positions 1 to 5. On each netting sample, four standard WHO cones were positioned over net swatches and secured in place using tape. Five laboratory-bred mosquitoes were introduced into each cone and exposed for 3 min, and four replicates were conducted per net piece (20 mosquitoes were exposed per net piece).

After each exposure, the mosquitoes were removed gently from the cones (by mouth aspiration), placed in paper cups and provided with cotton wool moistened with 10% sugar solution. Knockdown (KD60) was recorded after 60 min and mortality at 24, 48 and 72 h. Mosquitoes challenged to untreated nets were used as controls to monitor the quality of the bioassay. The bioassays and holding period were carried out at 27 ºC ± 2 ºC and 60%–100% relative humidity. Acceptable control mortality was ≤ 10% after 72 h holding time. Any tests exceeding the specified control cut off were repeated.

### WHO tunnel test

The tunnel tests were carried out from August to November 2020 according to standard WHO procedures [9]. Five 25 cm × 25 cm net pieces per ITN/control were cut adjacent to the swatches cut for cone assays from three nets per treatment arm to make a total of 15 pieces per study arm and to account for possible intra-net variability of insecticide loadings. Five tunnels were run with one sample from each treatment arm with one mosquito strain on a single night. Over 60 nights, all 15 pieces of ITNs per study arm were tested with four mosquito strains.

Non-blood-fed nulliparous females, 5–8 days old, sugar starved for 6–8 h were released in a 60-cm-long glass tunnel. At each end of the tunnel, a 25-cm square mosquito cage covered with polyester netting was fitted. At one third of the length, the netting sample was affixed. The surface of netting “available” to mosquitoes is 400 cm^2^ (20 cm × 20 cm), with 9 × 1-cm-diameter holes: one hole is located at the centre of the square; the other eight were equidistant and located 5 cm from the border. In the shorter section of the tunnel, a small rabbit, its back shaved and restrained in a mesh tunnel, was placed as bait (Fig. 1). In the cage at the end of the longer section of the tunnel, 100 female mosquitoes (one strain per replicate) were introduced at 21:00. The following morning at 09:00, the mosquitoes were removed using a mouth aspirator and counted separately from each section of the tunnel, and mortality and blood-feeding rates were recorded. The mosquitoes were placed in paper cups and provided with cotton wool moistened with 10% sugar solution. Mortality was recoded at 24, 48 and 72 h. Mosquitoes challenged to untreated nets were used as controls to monitor the quality of the bioassay. The bioassays and holding period were carried out at 27 ºC ± 2 ºC and 60%–100% relative humidity. Overall mortality was measured by pooling the mortalities of mosquitoes from the two sections of the tunnel. Acceptable feeding success and mortality in controls were 50% and 10%, respectively. Any tests not meeting the specified control cut-off were repeated.

### Ifakara ambient chamber test

The Ifakara ambient chamber test (I-ACT) experiments were conducted from August to September 2020 as described [16]. The study was performed using 10 experimental compartments with 10 volunteers, using two compartments (replicates) per treatment per night over 20 nights to give a total of 40 replicates per treatment arm. For each test, 60 nulliparous, sugar-starved, 5–8-day-old, laboratory-reared female mosquitoes were released per chamber with 15 mosquitoes per strain. Morphologically identical *Anopheles* mosquito species were marked with non-toxic fluorescent dye to distinguish them [20].

Male volunteers slept beneath the LN from 21.00 h to 09.00 h to represent user conditions and capture early morning circadian activity of mosquitoes. Each night, volunteers were rotated between chambers following a pre-prepared rotation schedule that was partially randomised. Each volunteer got into position beneath their net and released the mosquitoes within their compartment from holding cups. After the allotted experimental period, all mosquitoes within each of the compartments were removed by mouth aspiration. Each morning of the study, dead and resting mosquitoes were collected from inside the ITNs; dead mosquitoes were then collected from the floor of the chamber. Finally, resting mosquitoes were collected from the walls and roof of the chamber. Mosquitoes were sorted and scored by location as dead fed, dead unfed, alive fed and alive unfed, were held for 72 h at 27° ± 2 °C temperature and 60%–100% RH and provided with access to 10% sugar solution to assess delayed mortality. Acceptable feeding success and mortality in controls were 50% and 10%, respectively. Any tests not meeting the specified control cut-off were repeated.

### Experimental hut procedures

The evaluation was conducted from February to March 2021 in ten (10) experimental huts (Ifakara design) as described [21]. The dimensions of each hut are 3.25 m × 3.5 m × 2 m (*l* × *w* × *h*) with a gabled roof of 0.5 m apex and volume of 28.43 m^3^. The huts have 10-cm eave gaps with 10-cm baffles (to reduce mosquito egress to < 10%) on three sides and two window exit traps. The evaluation used ten male volunteers using two simultaneous partially randomized blocks of 5 × 5 Latin square design (Williams design) with volunteers and nets rotated to control for differences in mosquito densities due to volunteer kairomones and hut location. The study was conducted for 5 rounds over 25 nights using two huts per treatment per night. Data were collected for 5 nights and then huts were aired for 1 night before the next treatment was introduced into the huts. Sleepers were rotated sequentially among huts each night of the study using a pre-prepared roster. Before testing in the experimental huts, preliminary mosquito catches (with untreated nets) were performed for 2 nights for training purposes.

Volunteer sleepers entered the hut at 19.00 h and remained inside until 06.00 h. Each morning of the study, dead and resting mosquitoes were collected from inside the nets and exit traps using mouth aspirators, and from the floor, walls and roof of the hut using Prokopack aspirators [22]. Mosquitoes were sorted and scored by location as dead fed, dead unfed, alive fed and alive unfed, were held for 72 h at 27° ± 5 °C temperature and 40%–100% RH and provided with access to 10% sugar solution to assess delayed mortality. Acceptable 72-h mortality in controls was 10%.

### Mosquito retention

Immediately after the main experimental hut trial, a retention test was conducted by releasing wild *An. arabiensis* mosquitoes that were collected from the area by human landing catch (HLC). Fifteen mosquitoes marked with non-toxic fluorescent powder were released in each of the ten huts with the same treatment arms as the main experiment hut design. Five huts were completely closed while the other five were left with open eaves (with 10-cm baffles to reduce egress) and two window exit traps as per main experimental hut study. This experiment was conducted for 5 nights. Each morning mosquitoes were sorted, scored and held for 72 h to observe delayed mortality for each treatment arm.

### Impact of hut design on mosquito mortality estimates

To explore the effect of experimental hut design on the measured efficacy of the ITNs, two experiments were conducted between July and August 2021 using ten huts with the same five treatment arms as per the main experimental huts experiment. For the first experiment, conducted over 5 nights, windows were completely covered with white cloth to block light, ventilation and exit. For the remaining 25 nights, 5 huts had windows blocked with netting to allow light and ventilation but to block exit and five huts had exit traps as per main trial. Like the main experimental hut trial, the design was a fully balanced 5 × 5 Latin square with five huts per condition (window exit traps vs. netting windows). Each morning mosquitoes were sorted, scored and held for 72 h to observe delayed mortality for each treatment arm.

### Sample size and power

A sample size calculation for generalized linear mixed effects models (GLMMs) through simulation [23] in R statistical software 3.02 https://www.r-project.org/ was performed for the I-ACT and experimental huts. For the I-ACT, to detect a 10% effect difference between the nets, simulations were performed using an estimated mosquito mortality of 80% for unwashed Interceptor^®^ G2, 70% for unwashed Interceptor^®^ and 10% for SafiNet^®^ (deliberately holed). The design was for five arms replicated in two groups, with each volunteer testing each treatment one time over 20 replicates within its group (i.e. 40 replicates per arm), with an inter-observational variance of 0.42 for the night of observation based on the variance of the random effects observed in a previous study. The evaluation was powered at 81% for 15 mosquitoes of each strain released per chamber using 1000 simulations.

For experimental huts, simulation was performed using a Latin square design with volunteers rotating nightly and accounted for as a fixed effect for 25 nights of data collection in 10 huts. The study had 84% power to detect the difference between Interceptor^®^ G2 LN and Interceptor^®^ LN on mosquito mortality endpoints, with two huts per treatment arm (i.e. 50 replicates per arm). The study power was calculated based on a previous study of pyrethroid nets conducted in the same area, with the estimation of 20 *An. arabiensis* mosquitoes per night per hut, 21% mortality in unwashed Interceptor^®^ G2, 7% in 20× washed Interceptor^®^ G2 and 10% in unwashed Interceptor^®^ vs. 4% in 20 times washed and 1% in negative control, and overdispersion parameter for daily variation was set at intermediate 0.44.

To test whether I-ACT measures similarly to the WHO tunnels (H_0_:*m*2 = *m*1) a power calculation using Satterthwaite’s *t*-test was conducted in STATA 16 software (StataCorp LLC, College Station TX, USA) for two unpaired sample means assuming unequal variance. The power estimated was > 90% based on estimates from previous studies conducted in the same setting: mean mortality of 81.5% for WHO tunnel test with an assumed daily variation of 0.5 and 15 replicates per arm and mean mortality estimates of 86.5% and an assumed daily variation of 0.42 with 40 replicates per arm were considered for I-ACT.

### Statistical analysis

Data were entered and validated in Excel using double entry system and exported into STATA 16 software (StataCorp LLC, College Station TX, USA) for further cleaning and analysis. Descriptive statistics were used for data summarization, where arithmetic mean percentage of mosquito control corrected mortality at 72 h and arithmetic mean percentage blood-feeding inhibition for each test and species was presented. Control corrected mortality was calculated by using Abbott's formula: (treatment mortality − control mortality/(1 − control mortality)*100% and blood-feeding inhibition was calculated by taking the total number of unfed mosquitoes divided by total mosquito recapture per hut night [24].

Multivariable mixed-effect logistic regression with binomial error and log link was used to compare the performance of Interceptor^®^ G2 to Interceptor^®^ on the mortality and blood-feeding endpoints for each species in WHO cone (mortality only), tunnel, I-ACT and experimental huts. Fixed effects were treatment, volunteer, hut number/position (for experimental hut) and night of the experiment. The same regression was used for the comparison of hut design, whereby an interaction term between hut design and ITN type was also included in the model.

## Results

### Comparison of 72-h mortality in each bioassay

#### Resistant mosquitoes

**Fig. 2:**
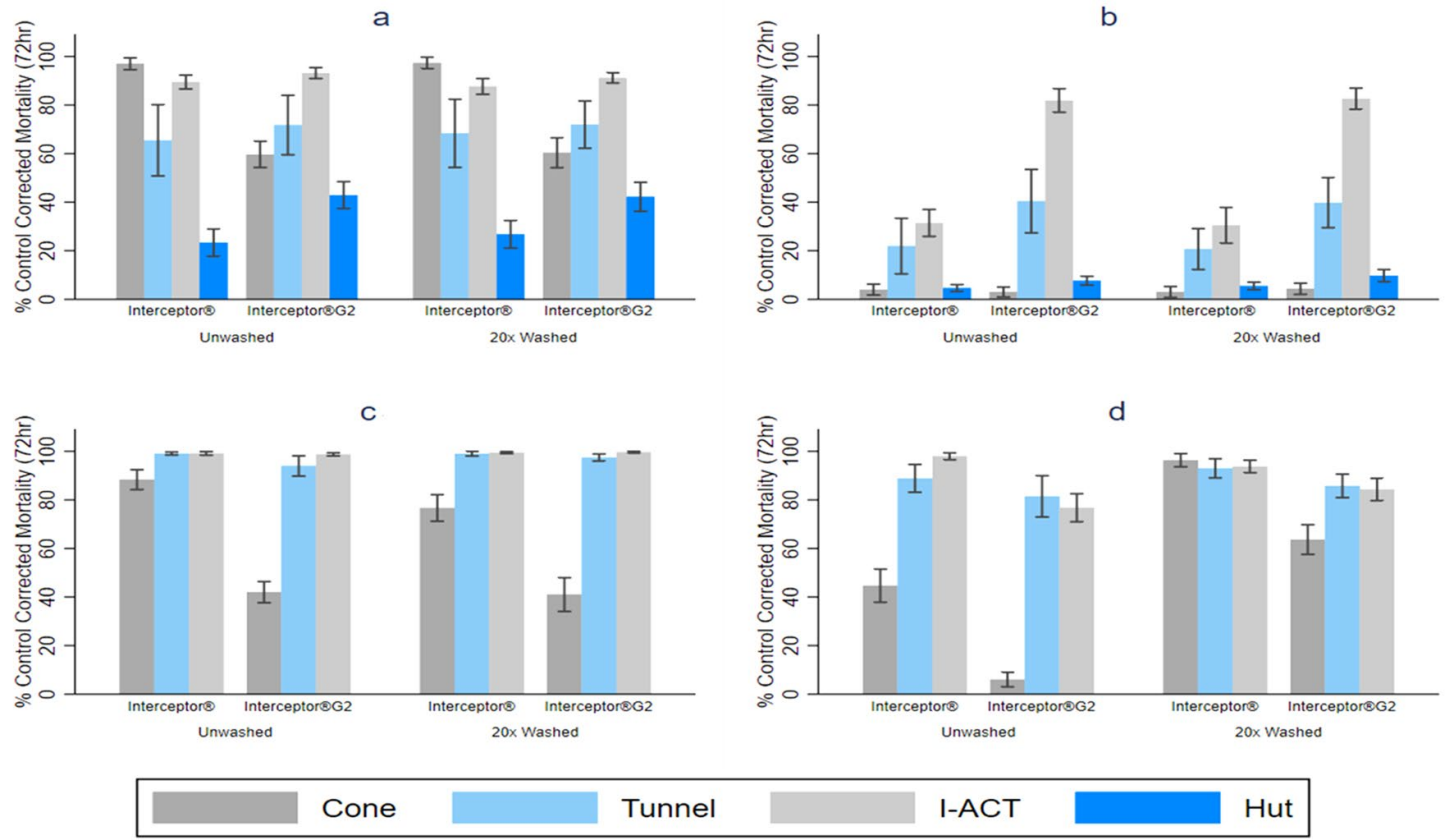
Mosquito mortality after exposure to Interceptor^®^ and Interceptor^®^ G2 ITNs in Ifakara ambient chamber test (IACT), WHO tunnel test, cone and experimental hut. **a** *An. arabiensis*, **b** *Cx. quinquefasciatus*, **c** *An. gambiae* s.s., **d** *Ae. aegypti*

The mortality of resistant mosquitoes was higher for Interceptor^®^ G2 compared to Interceptor^®^ ITN in all bioassays except WHO cone bioassays where the reverse result was seen (Fig. 2a, b, Table 2).

**Table 2:** Logistic regression analysis to compare mosquito mortality in Interceptor^®^ and Interceptor^®^ G2 ITNs after exposure in WHO cone and tunnel tests, Ifakara ambient chamber test and experimental hut, Tanzania

	WHO cone	Tunnel	I-ACT	Hut	Huts with netting window
	OR (95% CI)	OR (95% CI)	OR (95% CI)	OR (95% CI)	OR (95% CI)
*Anopheles arabiensis* (resistant)					
Overall					
Interceptor^®^	1	1	1	1	1
Interceptor^®^G2	0.04 (0.03, 0.07)	1.33 (1.17, 1.51)	1.55 (1.16, 2.07)	2.38 (2.09,2.72)	2.43 (1.88, 3.14)
Unwashed					
Interceptor^®^	1	1	1	1	1
Interceptor^®^G2	0.05 (0.20, 0.09)	1.42 (1.19, 1.70)	1.61 (1.05, 2.49)	2.53 (1.96,3.26)	2.55 (1.76, 3.68)
Washed, 20×					
Interceptor^®^	1	1	1	1	1
Interceptor^®^G2	0.04 (0.2, 0.09)	1.23 (1.03, 1.48)	1.50 (1.02, 2.23)	2.36 (2.02, 2.77)	2.32 (1.63, 3.31)
*Culex quinquefasciatus* (resistant)					
Overall					
Interceptor^®^	1	1	1	1	1
Interceptor^®^G2	1.05 (0.57, 1.93)*	2.55 (2.26, 2.55)	13.40 (10.75, 16.67)	1.50 (1.31, 1.73)	1.40 (1.19, 1.63)
Unwashed					
Interceptor^®^	1	1	1	1	1
Interceptor^®^G2	0.74 (0.31, 1.79)*	2.52 (2.13,3.00)	12.71 (9.43,17.14)	1.52 (1.23,1.88)	1.25 (1.00, 1.57)
Washed, 20×					
Interceptor^®^	1	1	1	1	1
Interceptor^®^G2	1.46 (0.62, 3.50)*	2.58 (2.17, 3.06)	14.11 (10.42, 19.11)	1.50 (1.25, 1.80)	1.55 (1.24, 1.94)
*An. gambiae* s.s*.* (susceptible)					
Overall					
Interceptor^®^	1	1	1		
Interceptor^®^G2	0.15 (0.11, 0.19)	0.21 (0.13, 0.32)	0.96 (0.34, 2.72)*	–	–
Unwashed					
Interceptor^®^	1	1	1		
Interceptor^®^G2	0.10 (0.06, 0.15)	0.14 (0.07, 0.25)	0.65 (0.18, 2.38)*	–	–
Washed, 20×					
Interceptor^®^	1	1	1		
Interceptor^®^G2	0.21 (0.15, 0.30)	0.36 (0.19, 0.69)	1.98 (0.30, 13.3)*	–	–
*Aedes aegypti* (susceptible)					
Overall					
Interceptor^®^	1	1	1		
Interceptor^®^G2	0.07 (0.05, 0.11)	0.21 (0.13, 0.32)	0.14 (0.10, 0.20)	–	–
Unwashed					
Interceptor^®^	1	1	1		
Interceptor^®^G2	0.08 (0.05, 0.13)	0.53 (0.43, 0.67)	0.05 (0.03, 0.10)	–	–
Washed, 20×					
Interceptor^®^	1	1	1		
Interceptor^®^G2	0.07 (0.03, 0.13)	0.43 (0.33, 0.57)	0.28 (0.18, 0.43)	–	–

Odds ratio adjusted for net type, volunteer, hut position/number and day of the experiment

Laboratory-reared mosquitoes were used for WHO cone, WHO tunnel and I-ACT

For the experimental hut trial, the ITNs were tested against free-flying wild pyrethroid-resistant *Anopheles arabiensis* and *Culex quinquefasciatus* in Lupiro, Ifakara

^*^p-value > 0.05

Mortality of *An. arabiensis* at 72 h for unwashed Interceptor^®^ G2 compared to unwashed Interceptor^®^ was 59.7% (95% CI: 54.2–65.1) vs. 97.0% (95% CI: 94.6–99.4) in WHO cone bioassays (OR = 0.05 [95% CI: 0.20–0.09], *p* < 0.001) showing superiority of Interceptor^®^ against resistant mosquitoes when measured in this way. For *Culex quinquefasciatus*, no significant difference was seen in unwashed Interceptor^®^ G2 compared to unwashed Interceptor^®^ in WHO cone: 3% (95% CI: 0.9–5.1) vs. 4.0% (95% CI: 1.7–6.3) (OR = 0.74 [95% CI: 0.31–1.79], *p* = 0.507).

For “free-flying assays” challenging *An. arabiensis*, Interceptor^®^ G2 was superior to Interceptor^®^ at 72-h mortality endpoints in all three bioassays (Fig. 2a, Table 2) with a similar magnitude of difference in odds ratios estimated in tunnel tests and I-ACT with unwashed Interceptor^®^ G2 compared to unwashed Interceptor^®^, WHO tunnel: 71.8% (95% CI: 59.5–84.0) vs. 65.5% (95% CI: 50.8–80.1), (OR = 1.42 [95% CI: 1.19–1.70], *p* < 0.001), I-ACT: 93.2% (95% CI: 90.9–95.4) vs. 89.5% (95% CI: 86.6–92.3), (OR = 1.61 [95% CI: 1.05–2.49], *p* = 0.031), and a greater magnitude of difference between the nets measured in experimental huts: 42.9% (95% CI: 37.3–48.5) vs. 23.3% (95% CI: 17.7–29.0), (OR = 2.53 [95% CI: 2.09–2.72], *p* < 0.001). The same trend was observed among the 20× washed nets.

For “free-flying assays” challenging *Cx. quinquefasciatus*, Interceptor^®^ G2 was superior to Interceptor^®^ at 72-h mortality endpoints in all three assays with unwashed Interceptor^®^ G2 compared to unwashed Interceptor^®^, WHO tunnel: 40.4% (95% CI: 27.3–53.5) vs. 21.9% (95% CI: 0.4–33.4), (OR = 2.52 [95% CI: 2.13–3.00], *p* < 0.001), I-ACT: 81.9% (95% CI: 77.0–86.7) vs. 31.4% (95% CI: 25.9–37.0), (OR = 12.71 [95% CI: 9.43–17.14], *p* < 0.001) and experimental hut: 7.7% (95% CI: 3.8, 9.5) vs. 4.6% (95% CI: 3.2–6.0), (OR = 1.52 [95% CI: 1.23–1.88], *p* < 0.001). The same trend was observed among the 20× washed nets (Fig. 2b, Table 2).

#### Susceptible mosquitoes

The 72-h mortality of susceptible mosquitoes was lower for Interceptor^®^ G2 than for Interceptor^®^ (Fig. 2c, d, Table 2) in all laboratory bioassays (the experimental hut site had wild resistant mosquitoes only). Mortality for *An. gambiae* in unwashed Interceptor^®^ G2 compared to unwashed Interceptor^®^ in the WHO cone test was: 42.2% (95% CI: 37.6–46.4) vs. 88.3% (95% CI: 84.2–92.4), (OR = 0.10 [95% CI: 0.06–0.15], *p* < 0.001). A similar pattern of lower mortality for Interceptor^®^ G2 was also seen among *Ae. aegypti* mosquitoes: 6.0% (95% CI: 3.0–9.0) vs. 44.7% (95% CI: 37.8–51.5), (OR = 0.08 [95% CI: 0.05–0.30], *p* < 0.001).

For “free-flying assays” unwashed Interceptor^®^ G2 showed lower 72-h mortality than unwashed Interceptor^®^ for *An. gambiae*. In both assays unwashed Interceptor^®^ G2 compared to unwashed Interceptor^®^, WHO tunnel was 94.0% (95% CI: 89.8–98.1) vs. 99.1% (95% CI: 98.5–99.7), (OR = 0.14 [95% CI: 0.07–0.25], *p* < 0.001) and I-ACT: 98.7% (95% CI: 98.1–99.4) vs. 99.1% (95% CI: 98.4–99.9) although this difference was marginal (OR = 0.65 [95% CI: 0.18–2.38], *p* = 0.519). Against susceptible *Ae. aegypti* for WHO tunnel the result was: 81.5% (95% CI: 73.0–90.0) vs. 88.9% (95% CI: 83.2–94.6) (OR = 0.53 [95% CI: 0.43–0.67], *p* < 0.001) and I-ACT: 76.8% (95% CI: 71.0–82.5) vs. 97.9% (95% CI: 96.5–99.3) (OR = 0.05 [95% CI: 0.03–0.10], *p* < 0.001). The same trend was observed among the 20× washed nets. The percentage mortality and mean difference between bioassays for all mosquito species are presented in Additional file 1: Table S1.

#### Mosquito blood-feeding in WHO tunnel, I-ACT and experimental hut

**Fig. 3:**
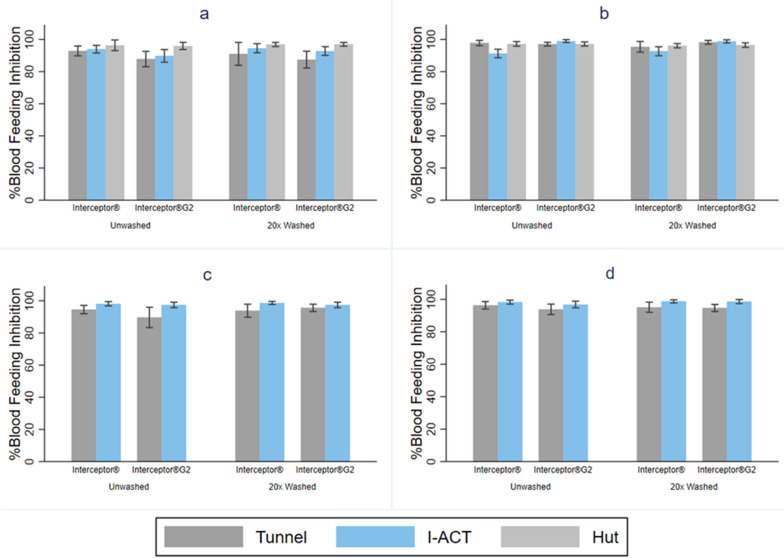
Mosquito blood-feeding inhibition after exposure to Interceptor and Interceptor^®^ G2 ITNs in Ifakara ambient chamber test (IACT), WHO tunnel test and experimental hut. **a** *An. arabiensis*, **b** *Cx. quinquefasciatus*, **c** *An. gambiae* s.s., **d** *Ae. aegypti*

For all bioassays and strains, blood-feeding inhibition was very high (Fig. 3, Table 3). Both Interceptor^®^ G2 and Interceptor^®^ gave high levels of blood-feeding inhibition in all “free-flying” bioassays.

**Table 3:** Logistic regression analysis to compare mosquito blood-feeding success in Interceptor and Interceptor G2 ITNs after exposure in WHO tunnel test, Ifakara ambient chamber test and experimental hut, Tanzania

	Tunnel	I-ACT	Hut	Huts with netting window
	OR (95% CI)	OR (95% CI)	OR (95% CI)	OR (95% CI)
*Anopheles arabiensis* (resistant)				
Overall				
Interceptor^®^	1	1	1	1
Interceptor^®^G2	1.75 (1.46, 2.09)*	1.53 (1.10, 2.13)*	1.04 (0.74, 1.47)	1.42 (0.80, 2.53)
Unwashed				
Interceptor^®^	1	1		1
Interceptor^®^G2	1.92 (1.48, 2.48)*	1.67 (1.07, 2.62)*	1.06 (0.54,2.10)	1.81 (0.79, 4.14)
Washed, 20×				
Interceptor^®^	1	1	1	1
Interceptor^®^G2	1.60 (1.24, 2.05)*	1.37 (0.83, 2.25)	1.09 (0.72, 1.63)	1.15 (0.53, 2.50)
*Culex quinquefasciatus* (resistant)				
Overall				
Interceptor^®^	1	1	1	1
Interceptor^®^G2	0.73 (0.53, 1.00)	0.13 (0.07, 0.23)*	1.34 (0.88, 2.04)	0.63 (0.49, 0.81)*
Unwashed				
Interceptor^®^	1	1		1
Interceptor^®^G2	1.36 (0.85, 2.17)	0.11 (0.05, 0.26)*	1.04 (0.76, 1.42)	0.67 (0.45, 1.02)
Washed, 20×				
Interceptor^®^	1	1	1	1
Interceptor^®^G2	0.41 (0.26, 0.65)*	0.14 (0.06, 0.33)*	1.11 (0.86, 1.42)	1.59 (0.43, 0.82)*
*An. gambiae* s.s. (susceptible)				
Overall				
Interceptor^®^	1	1		
Interceptor^®^G2	1.28 (1.03, 1.59)*	1.55 (0.86, 2.80)	–	–
Unwashed				
Interceptor^®^	1	1		
Interceptor^®^G2	1.95 (1.46, 2.61)*	1.41 (0.63, 3.17)	–	–
Washed, 20×				
Interceptor^®^	1	1		
Interceptor^®^G2	1.81 (1.35, 2.41)*	1.75 (0.69, 4.41)	–	–
*Aedes aegypti* (susceptible)				
Overall				
Interceptor^®^	1	1		
Interceptor^®^G2	1.46 (1.14, 1.87)*	1.63 (0.87, 3.04)	–	–
Unwashed				
Interceptor^®^	1	1		
Interceptor^®^G2	2.03 (1.40, 2.93)*	1.92 (0.87, 4.24)	–	–
Washed, 20×				
Interceptor^®^	1	1		
Interceptor^®^G2	1.10 (0.79, 1.54)	1.20 (0.43, 3.36)	–	–

Odds ratio adjusted for net type, volunteer, hut position/number and day of the experiment

Laboratory-reared mosquitoes were used for tunnel and I-ACT

For the experimental hut trial, the ITNs were tested against free-flying wild pyrethroid-resistant *Anopheles arabiensis* and *Culex quinquefasciatus* in Lupiro, Ifakara

^*^p-value < 0.05

#### Resistant mosquitoes

For pyrethroid-resistant *An. arabiensis*, blood-feeding inhibition was lower with Interceptor^®^ G2 than Interceptor^®^ in WHO tunnel: 87.8% (95% CI: 82.9- 92.7) vs. 92.9% (95% CI: 89.8–96.0) OR blood-fed = 1.92 [95% CI:1.48–2.48], *p* < 0.001 and I-ACT: 89.8% (95% CI: 85.8- 93.7) vs. 93.9% (95% CI: 91.5–96.3) (OR blood-fed = 1.67 [95% CI: 1.07–2.62], *p* = 0.024). There was no difference between the two products measured in experimental huts: 95.9% (95% CI: 93.7–98.2) vs. 96.3% (95% CI: 93.0–99.7) (OR blood-fed = 1.06 [95% CI: 0.54–2.10], *p* = 0.857). A similar trend was observed for the 20× washed nets.

Blood-feeding inhibition was similar for Interceptor^®^ G2 compared to Interceptor^®^ with *Cx. Quinquefasciatus* in WHO tunnel: 97.8% (95CI: 96.1–99.5) vs. 97.1% (95% CI: 95.9–98.3) (OR blood fed = 1.36 [95% CI: 0.85- 2.17], *p* = 0.196) and experimental huts: 97.2% (95% CI: 95.9–98.4) vs. 97.2% (95% CI: 95.8–98.7) (OR = 1.04 [95% CI: 0.76–1.42], *p* = 0.827), whilst in I-ACT the difference was significantly different: 99.0% (95% CI: 98.1–99.9) vs. 91.3% (95% CI: 88.6–93.9) (OR blood fed = 0.11 [95% CI: 0.05–0.26], *p* < 0.001). A similar trend was observed for the 20× washed nets (Fig. 3a, b, Table 3). The percentage blood-feeding inhibition and mean difference between bioassays for all mosquito species are presented in Additional file 1: Table S2.

#### Susceptible mosquitoes

For unwashed Interceptor^®^, blood-feeding inhibition was measured in tunnel and I-ACT and a similar trend was observed in susceptible mosquitoes as was seen in the resistant laboratory strains (Fig. 3c, d, Table 3). For susceptible *An. gambiae*, blood-feeding inhibition was lower with Interceptor^®^ G2 than for Interceptor^®^ WHO tunnel: 89.7% (95% CI: 83.3–96.1) vs. 94.5% (95% CI: 91.9–97.2), (OR blood fed = 1.95 [95% CI: 1.46, 2.61], *p* < 0.001) and similar in I-ACT: 97.4% (95% CI: 95.7–99.1) vs. 98.2% (95% CI: 96.8–99.5), (OR blood fed = 1.41 [95% CI: 0.63–3.17], *p* = 0.407. For susceptible *Ae. Aegypti* in WHO tunnel, results showed: 93.8% (95% CI:90.6–97.1) vs. 96.3% (95% CI: 94.0–98.7) (OR blood fed = 2.03 [95% CI: 1.40–2.93], *p* < 0.001) and in I-ACT: 96.8% (95% CI: 94.8–98.9) vs. 98.3% (95% CI:97.1–99.5) (OR blood fed = 1.92 [95% CI: 0.87–4.24], *p* = 0.107). As before, for both species a similar pattern was seen for the 20× washed nets.

#### Proportion of Interceptor^®^ and Interceptor^®^ G2 passing WHO thresholds

The cone bioassay gave expected results for Interceptor^®^ with most pieces passing even after 20 washes (Table 4). Very few Interceptor^®^ G2 pieces passed WHO thresholds in the cone test, while most passed when tested by WHO tunnel or I-ACT tests even after 20 washes (Table 4).

**Table 4:** Number and proportion of net pieces passing using each laboratory bioassay based on WHO criteria of 80% control corrected mortality, 95% knockdown in WHO cone test, 90% blood-feeding inhibition or 80% control corrected mortality in tunnel test

ITNs type	Number of net pieces passing		
	Cone (*N* = 60)	Tunnel ( *N* = 15)	I-ACT (*N* = 40)
*Anopheles arabiensis* (Kingani strain, resistant)			
Unwashed	*n* (%)	*n* (%)	*n* (%)
Interceptor^®^	59 (98)	11 (73)	38 (95)
Interceptor^®^ G2	22 (37)	13 (87)	38 (95)
Washed, 20×			
Interceptor^®^	59 (98)	12 (80)	36 (90)
Interceptor^®^ G2	21 (35)	10 (67)	38 (95)
*An. gambiae* s.s. (Kisumu strain, susceptible)			
Unwashed			
Interceptor^®^	50 (83)	15 (100)	40 (100)
Interceptor^®^ G2	2 (3)	14 (100)	40 (100)
Washed, 20×			
Interceptor^®^	38 (63)	15 (100)	40 (100)
Interceptor^®^ G2	10 (17)	15 (100)	40 (100)

The WHO criteria for tunnel were adopted for I-ACT. Whole nets were used for I-ACT

#### mosquito retention and control corrected mortality of *An. arabiensis* in normal Ifakara experimental huts and modified huts

**Fig. 4:**
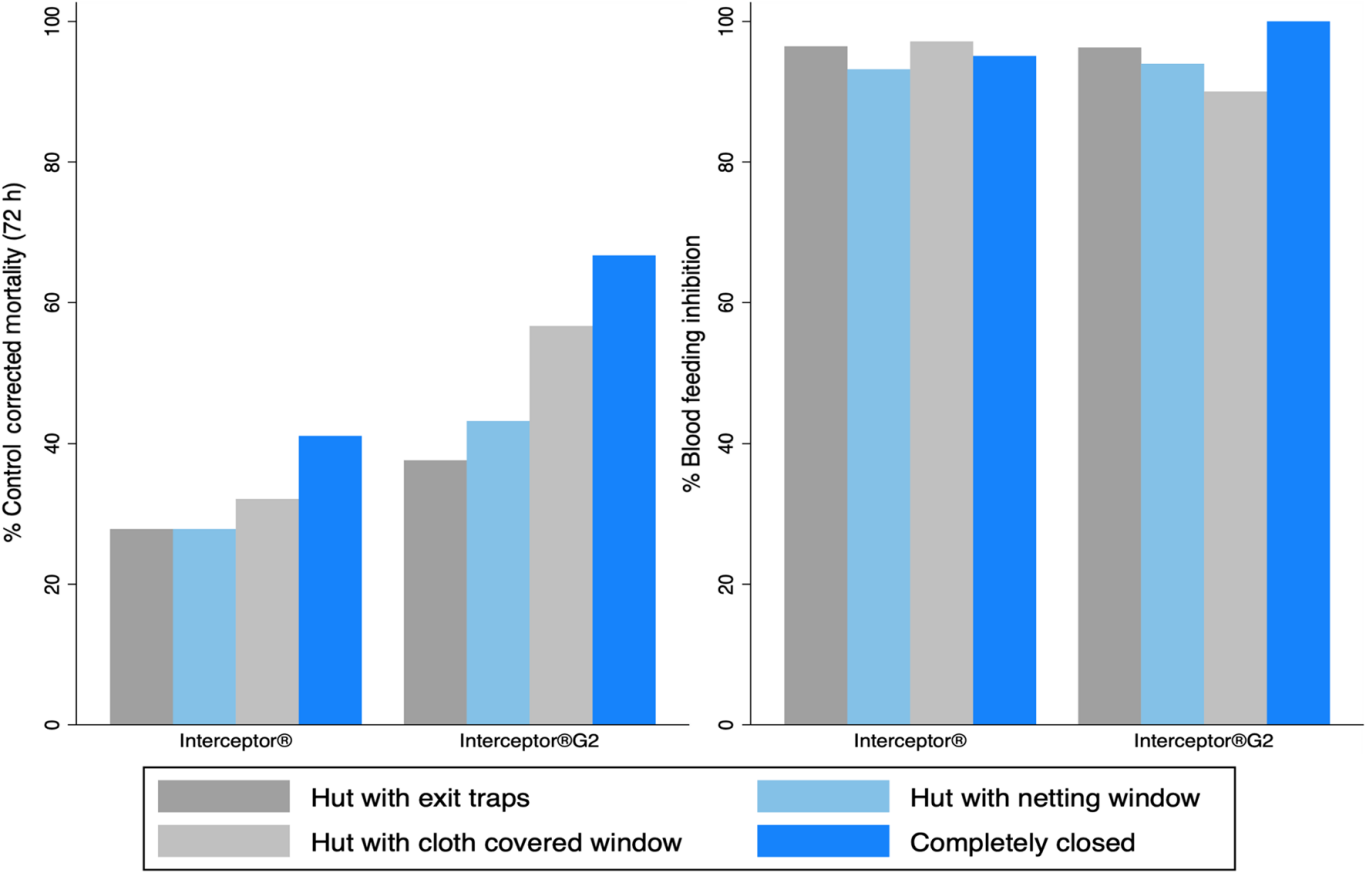
Comparison of mosquito mortality and blood-feeding inhibition of *An. arabiensis* in four different experimental hut designs in Ifakara, Tanzania

Mosquito retention was 88% (95% CI: 81.2–94.8) for completely closed huts and 89.8% (95% CI: 83.1–96.6) in the unmodified hut (with eave baffles and window exit traps). Mortality at 72 h among all treatment arms was higher when mosquitoes were prevented from leaving the hut in the completely closed huts (eaves and windows blocked), huts with cloth-covered windows and huts with netting-covered windows relative to the standard huts where mosquitoes were free to exit into window traps. In each of the four hut modifications, mortality was higher in the Interceptor^®^ G2 arm than in the Interceptor^®^ arm (Fig. 4).

Mortality at 72 h was highest in completely closed huts with eaves and window blocked with cloth, where light and ventilation were minimal: Interceptor^®^ G2 66.7% (95% CI: 47.1–86.3) and Interceptor^®^ 41.0% (95% CI: 21.1–61.1). Blocking the window with cloth and leaving opened eaves reduced 72-h mortality in Interceptor^®^ G2 by 56.7% (95% CI: 18.2–95.1) and Interceptor^®^ by 32.1% (95% CI: 0.00–78.8) arms, while blocking the window traps with netting to prevent egress while still allowing ventilation and light gave similar 72-h mortality at 27.9% (95% CI: 18.4–37.3) as the unmodified hut with exit traps at 27.9% (95% CI: 21.1–34.6) for Interceptor^®^ as well as Interceptor^®^ G2 at 43.1% (95% CI: 32.7–53.6) mortality with windows blocked with net and 37.6% (95% CI: 29.3–46.0) in the huts with exit traps (Fig. 4).

Preventing egress into window traps while still allowing ventilation was associated with 62% higher odds of 72-h mortality compared to the normal Ifakara huts (window with exit traps) (overall OR = l.62 [95% CI: 1.42–1.86]), *p* < 0.001). Modifying the hut design to prevent exit also increased the odds of blood-feeding overall (OR = 2.40 [95% CI: 1.73–3.21], *p* < 0.001). Increased odds of 72-h mortality and blood-feeding when egress was prevented were seen in both the Interceptor^®^ G2 arm mortality (OR = 1.99 [95% CI: 1.58–2.49], *p* < 0.001), blood-feeding OR = 2.54 [95% CI: 1.47–4.41], *p* < 0.001 and Interceptor^®^ arm mortality OR = 1.25 [95% CI: 1.01–1.54], *p* = 0.038, blood-feeding (OR = 1.73 [95% CI: 0.96–3.12], *p* = 0.070). A similar trend was observed for wild *Cx. quinquefasciatus* with the overall (OR = 1.65 [95% CI:1.48–1.84], *p* < 0.001) for mortality and (OR = 2.59 [95% CI: 2.22–3.02], *p* < 0.001) for blood-feeding. Interceptor^®^ arm showed: mortality (OR = 1.33 [95% CI: 1.12–1.58], *p* = 0.001), blood-feeding (OR = 2.47 [95% CI: 1.85–3.28], *p* < 0.001); Interceptor^®^ G2 arm (OR = 1.86 [95% CI: 1.59–2.17], *p* = 0.001), blood-feeding (OR = 1.50 [95% CI: 1.11- 2.03], *p* = 0.008).

When huts were modified with net-covered windows to prevent egress, the same trends in relative product performance were observed against both species. Interceptor^®^ G2 gave superior 72-h mortality (OR = 2.43 [95% CI: 1.88–3.14], *p* < 0.001) and similar blood-feeding inhibition (OR blood fed = 1.42 [95% CI:0.80–2.51], *p* = 0.226). In the *Cx. quinquefasciatus* arm Interceptor^®^ G2 gave superior 72-h mortality (OR = 1.40 [95% CI: 1.19–1.63], *p* < 0.001) and Interceptor^®^ gave superior blood-feeding inhibition (OR blood fed = 0.63 [95% CI: 0.49, 0.81], *p* < 0.001) (Tables 2, 3).

Comparison of mosquito mortality and blood-feeding success measured in two experimental huts is presented in Additional file 1: Table S3.

## Discussion

### Cone test results

The superiority of Interceptor^®^ G2 over Interceptor^®^ for 72-h mortality endpoints, which challenged metabolically resistant *An. arabiensis* and *Cx. quinquefasciatus*, was clearly seen in all “free-flying” bioassays but not in the WHO cone test where mosquitoes are unable to fly around and be metabolically active. This observation has been seen by several other authors [14, 25–27] leading to a consensus that the overnight tunnel test using a 72-h mortality endpoint is a superior laboratory bioassay for evaluation of chlorfenapyr [2, 28] relative to the cone test, which was designed to test contact insecticides that do not require metabolic conversion into a secondary metabolite (most ITN insecticides are classified as IRAC Group 3 sodium channel modulators). It has been routinely observed in experimental hut bioassays that Interceptor^®^ G2 gives greater mortality that Interceptor^®^ among pyrethroid-resistant mosquitoes [14, 25–27], while mosquitoes are foraging at night when their metabolic rate is high. Both the tunnel test and the I-ACT consistently predicted the superiority of Interceptor^®^ G2 over Interceptor^®^ observed in experimental hut tests.

The results of this study’s mortality elicited on *Cx. quinqufaciatus* from chlorfenapyr is consistent with other studies over the past decade [29, 30]. Preliminary investigations for chlorfenapyr intoxication in metabolically resistant *Cx. quinquefaciatus* in experimental huts by [31] demonstrated relatively high levels of control in both ITNs and for IRS in Benin. Later, [27] observed 3 × mortality with chlorfenapyr + alpha-cypermethrin-treated nets compared to pyrethroid-only nets demonstrating the effect of chlorfenapyr on free-flying exposures that optimize conversion rates of the pro-insecticide. However, it is also known that once ITNs develop holes, a pyrethroid treatment offers little or no protection against pyrethroid-resistant *Cu.quinquefaciatus* [32]. The relatively high level of control sustained in *Cx. quinqufaciatus* in this study and relatively good mortality are important resistance management attributes that make inclusion of chlorfenapyr into vector control interventions a significant advancement for active ingredient options.

### Use of I-ACT for durability monitoring

I-ACT is designed to be a bridging bioassay that reproduces a more natural interaction between the mosquito and human hosts beneath a bednet [16]. Blood-feeding inhibition and 72-h mortality measured by WHO tunnel and I-ACT were similar, meaning that the use of I-ACT for durability monitoring using WHO thresholds of 80% mortality and 90% feeding inhibition is appropriate. We suggest that I-ACT can be a useful alternative to tunnel tests for bioefficacy monitoring and upstream product evaluation as both the bioassays measure a similar odds ratio and I-ACT gives a precise estimation of bioefficacy because it uses laboratory-reared mosquitoes. This is particularly important when considering the longitudinal evaluation of product performance as occurs in durability bioefficacy evaluation for two reasons. Currently, experimental huts tests are being used to evaluate the insecticidal durability of Interceptor^®^ G2 [33]. It was been observed at all experimental hut testing sites that mosquito resistance to insecticides has intensified through time [34]. This needs to be factored into considerations about durability testing. ITN protection may wane more quickly against more resistant mosquito populations as nets age [35], and if older ITNs are tested 3 years after baseline efficacy has been calculated, it is possible that they will be tested against a more resistant mosquito population than that at baseline. Therefore, use of carefully maintained laboratory strains may be helpful to minimise differences in insecticide resistance levels. Second, a previous longitudinal durability trial of ITNs [17] using standard WHO cone and tunnel tests as well as I-ACT shows that ITNs are highly heterogeneous, with up to 100% variance after use (John Bradley personal communication), because of variable use practices (washing, drying, care, sleeping space) [36, 37] that result in differential levels of damage and insecticide content [38]. This fact, coupled with WHO/PQ accepted intra-net insecticidal manufacturing heterogeneities [39], shows the need to expedite testing modalities that improve comprehension of net performances by donor organisations, national malaria control programmes (NMCPs) and manufacturers alike.

### Thresholds are not a good idea in field trials

Mortality measured in the experimental hut is lower than in the WHO tunnel tests or I-ACT meaning that tests comparing products are appropriate rather than setting a threshold of efficacy. These study data support the WHO Pesticide Evaluation Scheme (WHOPES) efficacy criteria for phase II studies [9]. This is particularly important for field evaluation of public health tools because insecticide resistance involves highly complex mechanisms, genes and gene interactions [6] that are heterogeneous in time and space [40]. Insecticide resistance is modified by selection pressures from constantly changing environmental parameters, such as insecticide/pesticide usage in agriculture and biotic interactions with other organisms that affect both the overall mosquito responses to insecticides and the selection of resistance mechanisms [41]. However, in all comparisons tested in “free-flying” bioassays with resistant mosquito strains, it was possible to predict the superiority of Interceptor^®^ G2 over Interceptor^®^. Therefore, the use of a standard comparator net to provide performance benchmarks when evaluating the performance of new products is critical. The added value of a new product relative to pyrethroid-only nets has proven extremely useful when synthesising evidence for PBO nets [42].

### Impact of bioassay design on ITN evaluation

**Fig. 5:**
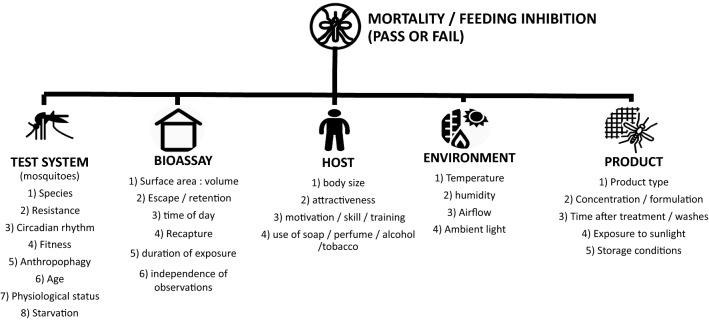
Factors determining bioassay results beyond product characteristics

Several bioassay design factors affect the efficacy of ITNs (Fig. 5). It was clearly seen that “free-flying” tests are appropriate for evaluation of chlorfenapyr as they predict the results of gold standard experimental hut tests. Use of a resistant strain is also clearly important since very little difference was seen between the products when susceptible strains were used, as has also been reported by several other authors [25, 27]. Ensuring that mosquitoes are metabolically active is an important factor when evaluating chlorfenapyr. The enzymatic transformation of parent chlorfenapyr (CL303630) to its pro-insecticidal metabolite (CL303268) is dependent on mosquito metabolism, a process that may take time to begin, but once conversion is started the insect’s respiration is increased [4] and it follows that this will favour additional conversion of the parent form to the potent metabolite. In nature, mosquitoes will encounter chlorfenapyr while foraging at night when their metabolic rate is high, which will increase the conversion of the parent to the metabolite, increasing intoxication. Bioassays at night and at higher temperature have clearly demonstrated that this is an essential consideration when measuring the bioefficacy of chlorfenapyr [14]. It is important to note that chlorfenapyr does indeed induce mortality at night in field tests when ambient temperatures are lower if they are actively foraging. The average temperature during I-ACT experiments at night was 24.6 (95% CI: 24.4–24.7) °C. However, the conversion rates from parent to active metabolite will be delayed until sufficient conversion has occurred. This is an important consideration for testing and may affect the level of mortality measured. Longer holding times may be appropriate at lower temperatures.

The differences in odds ratios measured in the experimental huts (2.28) and from the I-ACT (1.55) and tunnel tests (1.33) are likely due to the differences in mosquito activity, since wild mosquitoes would have flown into the experimental huts and been highly metabolically active, favouring the conversion of the parent to the metabolite and enhancing the difference in mortality observed between the Interceptor^®^ and Interceptor^®^ G2 ITNs. However, the difference in absolute mean mortality measured is probably due to the bioassay design influencing mosquito probability of encountering insecticide-treated surfaces (Fig. 1). The tunnel and I-ACT tests have a similar surface area (SA) to volume ratio, and therefore the likelihood of a mosquito encountering insecticide is more similar between these assays than in the experimental hut, which has a greater treated SA:volume ratio. The I-ACT also has a similar SA:volume ratio as East African Huts but a smaller ratio than that of West African huts (even when the verandah is included). The SA:volume ratio may also influence the probability of a mosquito blood-feeding. In tunnel tests where the host is presented very close to the mosquito release point, blood-feeding inhibition was lowest and blood-feeding inhibition increased as the area of the test arena increased.

The importance of increasing the probability of mosquito encounter with insecticides to increase absolute mortality is also illustrated by the hut modification experiments. By blocking mosquito exit points in experimental huts, which reduced cues such as light and air movement, mosquito 72-h mortality was almost doubled and blood-feeding was also increased, presumably because mosquitoes that could not escape continued to repeatedly host seek inside the huts. However, this increase in absolute values for the outcomes of interest, mortality and blood-feeding, needs to be balanced against the number of mosquitoes that enter the huts. It was observed that mosquito densities were substantially reduced when the window traps were blocked with cloth, presumably because it modified host odour plumes. Trials of ITNs and indoor residual spray (IRS) are powered on mosquito density as well as the relative difference in product performance [23], so it is important to allow adequate mosquito entry to confidently measure product performance. The advantage of having a large eave entry area has been seen in previous work in which West African huts caught fewer mosquitoes than Ifakara huts [43, 44], presumably because the West African huts have more limited airflow and smaller entry points. It is also interesting to note that the closed huts with mosquitoes released inside gave similar mortality estimates (67%) as the WHO tunnel (66%), as was also seen in the original validation of the tunnel test that measured 54% and 47% mortality in huts and tunnels, respectively, against a resistant strain with deltamethrin-treated ITNs [12].

Data collected in this trial agree with published data on Interceptor^®^ G2. Mortality at 72 h in the Ifakara experimental hut (40%) was similar to that in East African huts [14] that have exit traps. Mortality at 72 h in I-ACT (90%) was similar to that in West African huts [45] and the fully blocked Ifakara huts. In both Ifakara and East African hut designs mosquitoes are free to egress into exit traps and do not make repeated contacts with nets throughout the night. Conversely, in both the West African hut and I-ACT, mosquitoes cannot exit and are therefore more likely to make repeated contacts with the nets and sleeping humans. Hut design clearly influences both 72-h mosquito mortality and blood-feeding endpoints as seen in this study and others for *Anopheles* [34, 43, 44] and *Aedes* [46]. However, when considering the odds ratios, it was possible to detect differences between products with increased bioefficacy of Interceptor^®^ G2 compared to Interceptor^®^ in all hut designs, the tunnel test and the I-ACT, underlining the importance of comparing between products using well-powered studies with a rationale for the margins of acceptable difference between products [47].

### Selection of the correct strain for bioassay

The current study demonstrated that using the correct mosquito strain when evaluating ITNs is a critical consideration. The benefit of chlorfenapyr was only seen against the resistant strains, and against the susceptible strains Interceptor^®^ was more efficacious because it contains a higher dose of alphacypermethrin. Comparing between the products using both a susceptible and a resistant strain revealed the different modes of action of Interceptor^®^ and Interceptor^®^ G2.

### Considerations for each bioassay

Both I-ACT and experimental huts use the whole bednet and human host, which represent user conditions; however, there is no risk of disease for human participants in I-ACT because the mosquitoes used are laboratory reared. The WHO tunnel test is a well-established bioassay that has been shown to agree with experimental hut tests in this evaluation as well as others [12, 14]. However, it tests only a sample of net and is therefore only able to accurately measure the chemical durability of an ITN and not the chemical *and* physical durability. In addition, it requires a high number of mosquitoes (100 per replicate) and more testing days compared to I-ACT. In I-ACT there is possibility of testing more than one species or strain per chamber compared to tunnel test, which makes results more comparable. The I-ACT is a new assay that consistently predicts the results of experimental hut tests, measures with a similar magnitude of difference as a tunnel test and provides high-throughput and precise estimates of whole ITN protective efficacy in this study with chlorfenapyr as well as in previous studies with pyrethroid nets [17] at lower cost than tunnel tests. However, this method is yet to become a WHO/PQ-accepted testing modality despite the observed higher precision vs. current WHO-recommended modalities. The detailed descriptions of cost implications for each bioassay and how to build an I-ACT with the cost of establishment are described in Additional file 1: Table S4 and Additional file 2 respectively.

Laboratory and/or semi-field bioassays are not replacements for field evaluation, but they are useful if they can predict the results of field tests because they are substantially cheaper and more standardised. Experimental hut tests remain the gold standard test because they represent field conditions and can be related to public health impact [48]. However, variation in mosquito entrance in huts per night is highly heterogeneous and requires a high level of replication to achieve precision [23]. In addition, variation in hut designs affects outcome measurement [34] and should be considered when interpreting results. However, comparing between products, the same trends were consistently seen: Interceptor^®^ G2 was superior to Interceptor^®^ against resistant mosquitoes when they were tested in a “free-flying” scenario and Interceptor^®^ was superior to Interceptor^®^ G2 against susceptible strains, while the cone test was suitable for evaluating pyrethroids but not pro-insecticides such as chlorfenapyr.

## Conclusion

Interceptor^®^ G2 clearly demonstrated superior bioefficacy against resistant mosquitoes compared to Interceptor^®^ when mosquitoes were challenged in free-flying bioassays. The I-ACT measured similar odds ratios as the WHO tunnel, currently used for testing of ITNs with chlorfenapyr. Both free-flying laboratory bioassays (WHO tunnel and I-ACT) predicted the results of the experimental hut test. Experimental hut design has an influence on mosquito mortality; however, using the odds ratio, all free-flying tests gave consistent findings. In this setting, I-ACT was a reliable bioassay for bioefficacy testing of Interceptor^®^ G2 and may be a useful additional bioassay for durability monitoring of ITNs treated with pro-insecticides.

## Supplementary Information

**Additional file 1: Table S1.** Mosquito, control corrected mortality for laboratory mosquitoes after exposure to unwashed and washed Interceptor^®^ G2 ITNs in WHO tunnel, Ifakara ambient chamber test, tunnel and wild strain in experimental hut test. **Table S2.** Blood-feeding inhibition of laboratory mosquitoes after exposure to unwashed and washed Interceptor^®^ G2 ITNs in WHO tunnel, Ifakara ambient chamber test, tunnel and wild strain in experimental hut test. **Table S3.** Comparison of mosquito mortality and blood-feeding success in two hut designs, Ifakara, Tanzania. **Table S4.** Cost-effectiveness of WHO tunnel test, Ifakara ambient chamber test and experimental hut test in Tanzania.

**Additional file 2: Fig. S1.** A diagram on how to build the Ifakara ambient chamber test (I-ACT) and the cost to establish it.

**Additional file 3: Dataset S1.** Data analysed.

## Data Availability

All data generated or analysed during this study are included in this published article and its Additional file 3.

## Abbreviations

I-ACT: Ifakara ambient chamber test
ITN: Insecticide-treated net
NMCP: National Malaria Control Program
