# Supplementary material for: Influence of testing modality on bioefficacy for the evaluation of Interceptor® G2 mosquito nets to combat malaria mosquitoes in Tanzania
Source: Parasit Vectors. 2022 Apr 11;15:124. doi: 10.1186/s13071-022-05207-9 (PMC8996609; doi:10.1186/s13071-022-05207-9)
Supplement: Supplementary file 1 — Additional file 1: Table S1. Mosquito, control corrected mortality for laboratory mosquitoes after exposure to unwashed and washed Interceptor® G2 ITNs in WHO tunnel, Ifakara ambient chamber test, tunnel and wild strain in experimental hut test. Table S2. Blood-feeding inhibition of laboratory mosquitoes after exposure to unwashed and washed Interceptor® G2 ITNs in WHO tunnel, Ifakara ambient chamber test, tunnel and wild strain in experimental hut test. Table S3. Comparison of mosquito mortality and blood-feeding success in two hut designs, Ifakara, Tanzania. Table S4. Cost-effectiveness of WHO tunnel test, Ifakara ambient chamber test and experimental hut test in Tanzania. [file 13071_2022_5207_MOESM1_ESM.docx]

Table 1: Mosquito, control corrected mortality of laboratory reared mosquitoes measured 72 hours after exposure to unwashed and washed Interceptor® and Interceptor® G2 ITNs in WHO tunnel, Ifakara Ambient Chamber Test (I ACT) tunnel and wild strain in experimental hut test. The mean difference between methods is presented to show how methods differ in absolute mortality recorded

| Species | Test item | % 72hr Control corrected mortality (95% CI) | | | Mean difference between methods (95%CI) | | |
| --- | --- | --- | --- | --- | --- | --- | --- |
|  |  | Experimental hut* | I ACT | WHO Tunnel | I ACT vs Experimental hut | I ACT vs Tunnel | Tunnel vs  Experimental hut |
| Anopheles arabiensis (Kingani, resistant) | Interceptor®, unwashed | 23.3 (17.7, 29.0) | 89.5 (86.6, 92.3) | 65.5 (50.8, 80.1) | 66.2 (59.5, 72.8) | 23.9 (13.7, 34.2) | 42.2 (29.1, 55.3) |
|  | Interceptor® G2, unwashed | 42.9 (37.3, 48.5) | 93.2 (90.9, 95.4) | 71.8 ( 59.5, 84.0) | 50.3 (43.7, 56.8) | 21.4 (12.9, 29.9) | 28.8 (16.5, 41.2) |
|  | Interceptor®, washed 20x | 26.7 (21.0, 32.5) | 87.7 (84.4, 90.9) | 68.3 (54.3, 82.4) | 60.9 (53.9, 67.9) | 19.3 (09.1, 29.5) | 41.6 (28.6, 54.6) |
|  | Interceptor® G2, washed 20x | 42.2 (36.2, 48.2) | 91.3 (89.1, 93.3) | 71.9 (62.2, 81.6) | 48.9, (42.0, 55.9) | 19.3 (12.3, 26.2) | 29.7 (17.5, 41.9) |
| An. gambiae s.s. (Kisumu, susceptible) | Interceptor®, unwashed | - | 99.1 (98.4, 99.9) | 99.1 (98.5, 99.7) | - | 0.0 (-1.3, 1.3) |  |
|  | Interceptor® G2, unwashed |  | 98.7 (98.1, 99.4) | 94.0 (89.8, 98.1) |  | 4.7(2.0, 7.5) |  |
|  | Interceptor®, washed 20x |  | 99.5 (99.1,99.9) | 99.0 (98.1, 99.9) |  | 0.5 (-0.4, 1.4) |  |
|  | Interceptor® G2, washed 20x |  | 99.6 (99.3, 100) | 97.4 (96.0, 98.9) |  | 2.2 (1.1, 3.2) | - |
| Aedes aegypti (Bagamoyo, susceptible) | Interceptor®, unwashed | - | 97.9 (96.5, 99.3) | 88.9 ( 83.2, 94.6) | - | 9.1 (4.8, 13.3) |  |
|  | Interceptor® G2, unwashed |  | 76.8 (71.0, 82.5) | 81.5 ( 73.0, 90.0) |  | -4.7 (-15.7, 6.3) |  |
|  | Interceptor®, washed 20x |  | 93.8 (91.2, 96.3) | 93.0 ( 89.1, 96.9) |  | 0.8 (-4.2, 5.7) |  |
|  | Interceptor® G2, washed 20x |  | 84.3 (79.7, 88.9) | 85.8 (81.0, 90.6) |  | -1.5 (-9.7, 6.7) | - |
| Culex quinquefasciatus (Bagamoyo, resistant) | Interceptor®, unwashed | 4.6 (3.2, 6.0) | 31.4 (25.9, 37.0) | 21.9 (10.4, 33.4) | 26.8 (21.5, 32.1) | 9.5 (-2.1, 21.2) | 17.3 (10.4, 24.1) |
|  | Interceptor® G2, unwashed | 7.7 (3.8, 9.5) | 81.9 (77.0, 86.7) | 40.4 (27.3, 53.5) | 74.2 (69.4, 79.0) | 41.5 (30.0, 52.9) | 32.7 (24.8, 40.6) |
|  | Interceptor®, washed 20x | 5.5 (3.9, 7.0) | 30.5 (23.1, 37.8) | 20.7 (12.2, 29.1) | 24.9 (17.9, 31.9) | 9.8 (-3.6, 23.2) | 15.2 (9.7, 20.7) |
|  | Interceptor® G2, washed 20x | 9.7 (7.2, 12.1) | 82.6 (78.3, 86.9) | 39.8 ( 29.5, 50.1) | 72.9 (67.9, 77.7) | 42.8 (33.1, 52.5) | 30.1 (22.7, 37.4) |

*For the experimental hut trial, the LNs were tested against free flying wild pyrethroid resistant *Anopheles arabiensis* and Culex *quinquefasciatus* in Lupiro, Ifakara in Ifakara Experimental Huts

| Species | Test item | % BF inhibition (95%CI) | | | Mean difference between methods (95%CI) | | |
| --- | --- | --- | --- | --- | --- | --- | --- |
|  |  | Experimental hut* | I ACT | WHO Tunnel | I ACT vs Experimental hut | I ACT vs Tunnel | Tunnel vs  Experimental hut |
| Anopheles arabiensis (Kingani, resistant) | Interceptor®, unwashed | 96.3 (93.0, 99.7) | 93.9 (91.5, 96.3) | 92.9 (89.8, 96.0) | -2.4 (-6.7, 1.8) | 1.0 (-3.3, 5.4) | -3.5 (-9.7, 2.7) |
|  | Interceptor® G2, unwashed | 95.9 (93.7, 98.2) | 89.8 (85.8, 93.7) | 87.8 (82.9, 92.7) | -6.2 (-10.5, -1.8) | 1.9 (-5.2, 9.2) | -8.1 (-13.1, -3.2) |
|  | Interceptor®, washed 20x | 96.9 (95.6, 98.2) | 94.5 (91.7, 97.4) | 91.0 (83.8, 98.3) | -2.4 (-5.3, 0.6) | 3.5 (-2.9, 9.9) | -5.9 (-10.4, -1.3) |
|  | Interceptor® G2, washed 20x | 97.0 (95.8, 98.1) | 92.8 (90.1, 95.5) | 87.5 (82.2, 92.7) | -4.2 (-6.9, -1.4) | 5.3 (-0.2, 10.9) | -9.5 (-13.1, -5.9) |
| An. gambiae s.s. (Kisumu, susceptible) | Interceptor®, unwashed | - | 98.2 (96.8, 99.5) | 94.5 (91.9, 97.2) | - | 3.6 (0.9, 6.4) |  |
|  | Interceptor® G2, unwashed |  | 97.4 (95.7, 99.1) | 89.7 (83.3, 96.1) |  | 7.7 (2.9, 12.5) |  |
|  | Interceptor®, washed 20x |  | 98.7 (97.7, 99.6) | 93.9 (89.8, 97.9) |  | 4.8 (1.9, 7.8) |  |
|  | Interceptor® G2, washed 20x |  | 97.4 (95.7, 99.1) | 95.6 (93.3, 97.9) |  | 1.8 (-1.4, 4.9) | - |
| Aedes aegypti (Bagamoyo, susceptible) | Interceptor®, unwashed | - | 98.3 (97.1, 99.5) | 96.3 (94.0, 98.7) | - | 2.0 (-0.5, 4.5) |  |
|  | Interceptor® G2, unwashed |  | 96.8 (94.8, 98.9) | 93.8 (90.6, 97.1) |  | 3.0 (-1.0, 7.0) |  |
|  | Interceptor®, washed 20x |  | 98.8 (97.8, 99.7) | 95.1 (92.0, 98.3) |  | 3.6 (1.1, 6.1) |  |
|  | Interceptor® G2, washed 20x |  | 98.7 (97.4, 99.9) | 94.7 ( 92.5, 96.9) |  | 4.0 (1.5, 6.5) | - |
| Culex quinquefasciatus (Bagamoyo, resistant) | Interceptor®, unwashed | 97.2 (95.8, 98.7) | 91.3 (88.6, 93.9) | 97.8 (96.1, 99.5) | -5.9 (-8.9, -3.1) | -6.5 (-11.5, -2.0) | 0.6 (-2.3, 3.5) |
|  | Interceptor® G2, unwashed | 97.2 (95.9, 98.4) | 99.0 (98.1, 99.9) | 97.1 (95.9, 98.3) | 1.8 (0.2, 3.5) | 1.9 (0.3, 3.6) | -0.1 (-2.6, 2.4) |
|  | Interceptor®, washed 20x | 96.1 (94.8, 97.4) | 92.6 (89.8, 95.4) | 95.4 (92.1, 98.8) | -3.5 (-6.4, -0.5) | -2.8 (-7.9, 2.3) | -0.7 (-3.8, 2.4) |
|  | Interceptor® G2, washed 20x | 96.5 (95.1, 97.9) | 98.8 (97.8, 99.8) | 98.2 (97.0, 99.4) | 2.3 (0.5, 4.1) | 0.6 (-1.2, 2.3) | 1.7 (-0.9, 4.4) |

Table 2: Mosquito blood feeding inhibition of laboratory reared mosquitoes after exposure to unwashed and washed Interceptor® and Interceptor® G2 ITNs in WHO tunnel, Ifakara Ambient Chamber Test (I ACT) tunnel and wild strain in experimental hut test. The mean difference between methods is presented to show how methods differ in absolute reduction in blood feeding recorded

*For the experimental hut trial, the LNs were tested against free flying wild pyrethroid resistant *Anopheles arabiensis* and *Culex quinquefasciatus* in Lupiro, Ifakara in Ifakara Experimental Huts

Table 3: Logistic regression analysis to compare relative mosquito mortality and blood feeding success

in Interceptor® and Interceptor® G2 ITNs arms measured in two experimental huts with window exit traps and

without window exit traps (traps blocked by netting so that ventilation is not modified) in Ifakara, Tanzania (N=50)

|  | 72 hour Mortality | | Blood feeding | |
| --- | --- | --- | --- | --- |
|  | window exit trap | covered window | window exit trap | covered window |
|  | OR (95% CI) | OR (95% CI) | OR (95% CI) | OR (95% CI) |
| *Anopheles arabiensis* |  |  |  |  |
| Overall |  |  |  |  |
| Interceptor® | 1 | 1 | 1 | 1 |
| Interceptor®G2 | 1.20 (0.97, 1.49) | 2.43 (1.88, 3.14)* | 1.04 (0.53, 2.04) | 1.42 (0.80, 2.53) |
| *Culex quinquefasciatus* |  |  |  |  |
| Overall |  |  |  |  |
| Interceptor® | 1 | 1 | 1 | 1 |
| Interceptor®G2 | 1.19 (0.99, 1.44) | 1.40 (1.19, 1.63)* | 1.00 (0.74, 1.49) | 0.63 (0.49, 0.81)* |

*Note: Odds ratio adjusted for net type, volunteer, hut position and day of the experiment as fixed effect.*

**p-value <0.05*

Table 4: Cost effectiveness for WHO tunnel test, Ifakara Ambient Chamber Test and experimental hut test in Tanzania

|  | **Current study** | | | **2 arm Durability study** | | | |
| --- | --- | --- | --- | --- | --- | --- | --- |
|  | WHO Tunnel | I-ACT | Hut | WHO Tunnel | I-ACT | Hut  (high density | Hut  (Low density) |
| Number of days | **60** | **20** | **25** | **48** | **40** | **50** | **75** |
| Species per replicate | 1 | 4 | 1 | 1 | 2 | 1 | 1 |
| Number of Volunteers/Animal Baits per day | 5 | 10 | 10 | 3 | 10 | 10 | 10 |
| Number of Chambers/tunnels/huts per day | 5 | 10 | 10 | 3 | 10 | 10 | 10 |
| Number of mosquito per chamber per night | 100 | 60 | 20 | 100 | 30 | 20 | 5 |
|  | 100/species | 15/species | wild | 100/species | 15/species | wild | wild |
| Mosquito costs per experimental night ($1 fully costed) | 500 | 600 | NA | 300 | 270 | NA | NA |
| **Mosquito costs per experiment (USD)** | **30,000** | **12,000** | **NA** | **28,800** | **14,500** | **NA** | **NA** |
| Percent recapture | 100 | 99 | Retention=  89.8% | 100 | 99 | Retention~  89% | Retention~  89% |
| Malaria screen weekly | No | Yes | Yes | No | Yes | Yes | Yes |
| **Prophyaxis with doxycycline, screening (rapid diagnostic test) per experiment** | **-** | **30** | **35** | **-** | **105** | **140.4** | **140.5** |
| Veterinarian, animal husbandry, facility maintenance per annum | 6,000 | 1,000 | 11,000 | 6,000 | 1,000 | 11,000 | 11,000 |
| **facility costs (USD)** | **986** | **55** | **753** | **789** | **74** | **1085** | **2170** |
| Volunteer cost per person per replicate | - | 10 | 10 | - | 10 | 10 | 10 |
| **Volunteer costs per experiment (USD)** | **-** | **2,000** | **2,500** | **-** | **4,860** | **6,480** | **12,960** |
| Supervision costs per person per replicate | - | 50 | 50 | - | 50 | 50 | 50 |
| **Supervision costs per experiment (USD)** | **-** | **1,000** | **5,000** | **-** | **1,350** | **7,200** | **14,400** |
| **Total costs including 10% of facility costs (USD)** | **30,986** | **15,085** | **8,288** | **29,589** | **20,889** | **14905.4** | **29670.5** |

*Note: As the animal bait (Rabit) were alternated, more than the indicated number are needed*
