# Supplementary material for: Influence of testing modality on bioefficacy for the evaluation of Interceptor® G2 mosquito nets to combat malaria mosquitoes in Tanzania
Source: Parasit Vectors. 2022 Apr 11;15:124. doi: 10.1186/s13071-022-05207-9 (PMC8996609; doi:10.1186/s13071-022-05207-9)
Supplement: Supplementary file 2 — Additional file 2: Fig. S1. A diagram on how to build the Ifakara ambient chamber test (I-ACT) and the cost to establish it. [file 13071_2022_5207_MOESM2_ESM.docx]

| Quantity | Unit | Description | Unit price ($) | Total USD |
| --- | --- | --- | --- | --- |
| 1 | Item | **Substructure foundation and platform** |  |  |
| 175m | m | Excavation and concrete base layer | 5.19 | 908.25 |
| 175m/2 | m/2 | Building foundation walls with 6" concrete block | 12.98 | 2,272.00 |
| 4 | Rolls | Steel reinforcement (roll = 22m x 4m) | 160.00 | 640.00 |
| 151 | Tonne | compacted soil and hardcore stones | 20.00 | 3,020.00 |
| 22.5 | m/3 | Concrete slab 225m/2 x 100 mm | 121.00 | 2,725.00 |
| 225 | m/2 | concrete floor scread 5 cm thick | 5.17 | 1,168.00 |
| 150 | meter | water proof concrete ant channel | 9.69 | 1,454.00 |
| 40 | piece | Iron brackets for mounting roof pillars | 13.00 | 520.00 |
| 1 | Item | **Roof construction** |  |  |
| 120 | piece | Treated timber for pillars 6"x2" x 6m | 13.85 | 1,662.00 |
| 90 | piece | Treated timbers for roof trusses 4" x 2" x 6m | 10.39 | 935.00 |
| 92 | piece | Treated timbers for roof trusses 3" x 2" x 6m | 7.78 | 716.00 |
| 250 | meter | Heat reflecting foil | 9.52 | 2,379.00 |
| 270 | m/2 | Corrugated Iron sheets | 9.73 | 2,628.00 |
| 1 | Item | **Iron frames for iterior** |  |  |
| 92 | piece | Square tube 25mm x 25mm x 2mm (6m) | 18.17 | 1,672.00 |
| 12 | piece | square tube 30mm x 30mm x 2mm (6m) | 29.67 | 356.00 |
| 1 | piece | Steel plate 120cm x 240cm x 3mm | 121.00 | 121.00 |
|  |  | **Netting doors and chambers** |  |  |
| 420 | meter | Phizer fiberglass uv resistant netting 1.2m wide sewn | 7.14 | 3,000.00 |
| 10 | piece | Cloth and net chamber 3.5m x 2.3m x 2m | 216.00 | 2,160.00 |
| 70 | meter | White PVC material for doors and dividers | 10.00 | 700.00 |
|  |  | **Labour and transport** |  | 9,300.00 |
|  |  |  |  | **38,336.25** |
|  |  | VAT |  | 6,900.00 |
|  |  | **Total** |  | **45,236.25** |

| 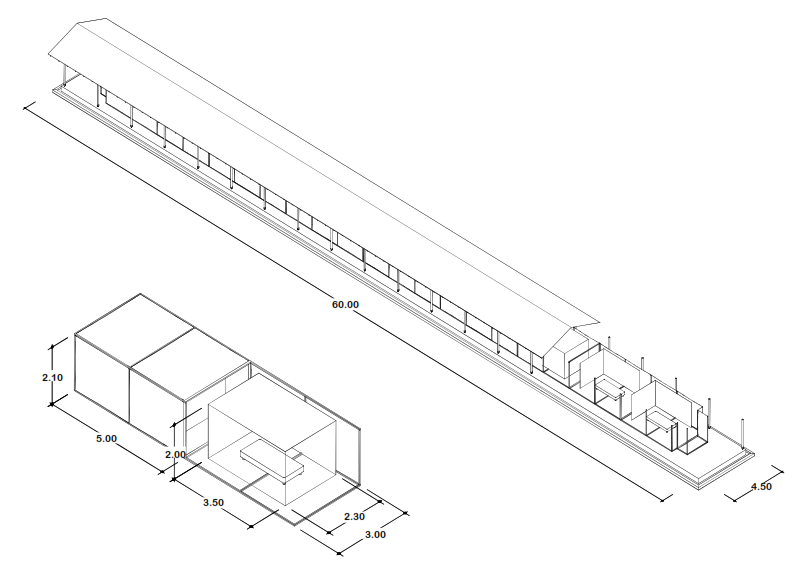 |
| --- |

For additional information and building plans, please contact jmoore@ihi.or.tz
